# How to make an oscillator

**DOI:** 10.7554/eLife.12260

**Published:** 2015-12-10

**Authors:** Bas JHM Rosier, Tom FA de Greef

**Affiliations:** Department of Biomedical Engineering and Institute for Complex Molecular Systems, Eindhoven University of Technology, Eindhoven, The Netherlands; Department of Biomedical Engineering and Institute for Complex Molecular Systems, Eindhoven University of Technology, Eindhoven, The Netherlandst.f.a.d.greef@tue.nl

**Keywords:** synthetic biology, genetic networks, oscillators, cell-free prototyping, *E. coli*

## Abstract

A cell-free approach reveals how genetic circuits can produce robust oscillations of proteins and other components.

**Related research article** Niederholtmeyer H, Sun ZZ, Hori Y, Yeung E, Verpoorte A, Murray RM, Maerkl SJ. 2015. Rapid cell-free forward engineering of novel genetic ring oscillators. *eLife* **4**: e09771. doi: 10.7554/eLife.09771

**Image** Circuits made of three or five genes can generate robust oscillations in the fluorescence of bacteria cells.

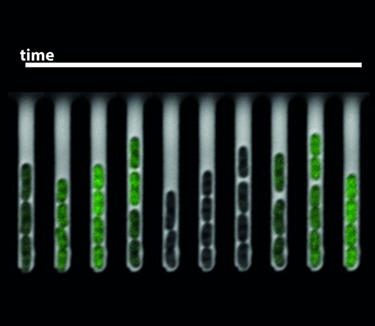

Ever since it was discovered that the level of calcium ions inside a cell can oscillate (Woods et al., 1986), biologists have been intrigued by the periodic nature of many cellular signals. While we are slowly starting to grasp the many and varied roles that these periodic oscillations play in cellular communication, an open question remains: how are networks of genes able to generate sustained oscillations? Now, in eLife, Sebastian Maerkl and co-workers – including Henrike Niederholtmeyer and Zachary Sun as joint first authors – report that they have used a synthetic biology approach to reveal how simple gene circuits can produce robust oscillations in cells (Niederholtmeyer et al., 2015).

Initially, it was argued that periodic oscillations in the level of calcium ions and other cellular components had no role in signaling, but decades of research has revealed that periodic signals are better at relaying information than non-periodic signals (Rapp, 1987; Behar and Hoffman, 2010; Purvis and Lahav, 2013; Levine et al., 2013). Both types of signal can encode information in the size (amplitude) of the signal, but the frequency and phase of periodic signals can also encode information. As a result, periodic signals may be able to regulate complex cell processes more precisely than non-periodic signals. Importantly, recent advances in single-cell analysis and optogenetics have resulted in numerous in-depth studies that reveal how critical events, such as the determination of cell fate and multicellular communication, are controlled by periodic signals (the review by Sonnen and Auleha, 2014 describes other examples).

Mathematical analysis shows that an essential element of an oscillating circuit is an inhibitory feedback loop: if the activity of one gene in such a feedback loop increases, it activates other genes in the circuit that ultimately inhibit it (Rapp, 1987; Novak and Tyson, 2008; Purcell et al., 2010). This feedback loop needs to have an in-built time delay to enable the activities of the genes in the circuit to fluctuate in regular cycles.

The rise of synthetic biology has made it possible to design and construct synthetic networks in living cells that perform a specific role. In an early example of this, researchers at Princeton reported that they had constructed an oscillatory gene network in *E. coli* based on a cyclic network of three genes called the repressilator (Elowitz and Leibler, 2000; Figure 1). Theory predicts that the repressilator and other ring oscillators that have an odd number of genes (nodes) should be capable of producing sustained oscillations. However, since designing, building and testing new gene networks in living cells is a lengthy process, ring oscillators with more than three nodes have not been reported.

**Figure 1.**
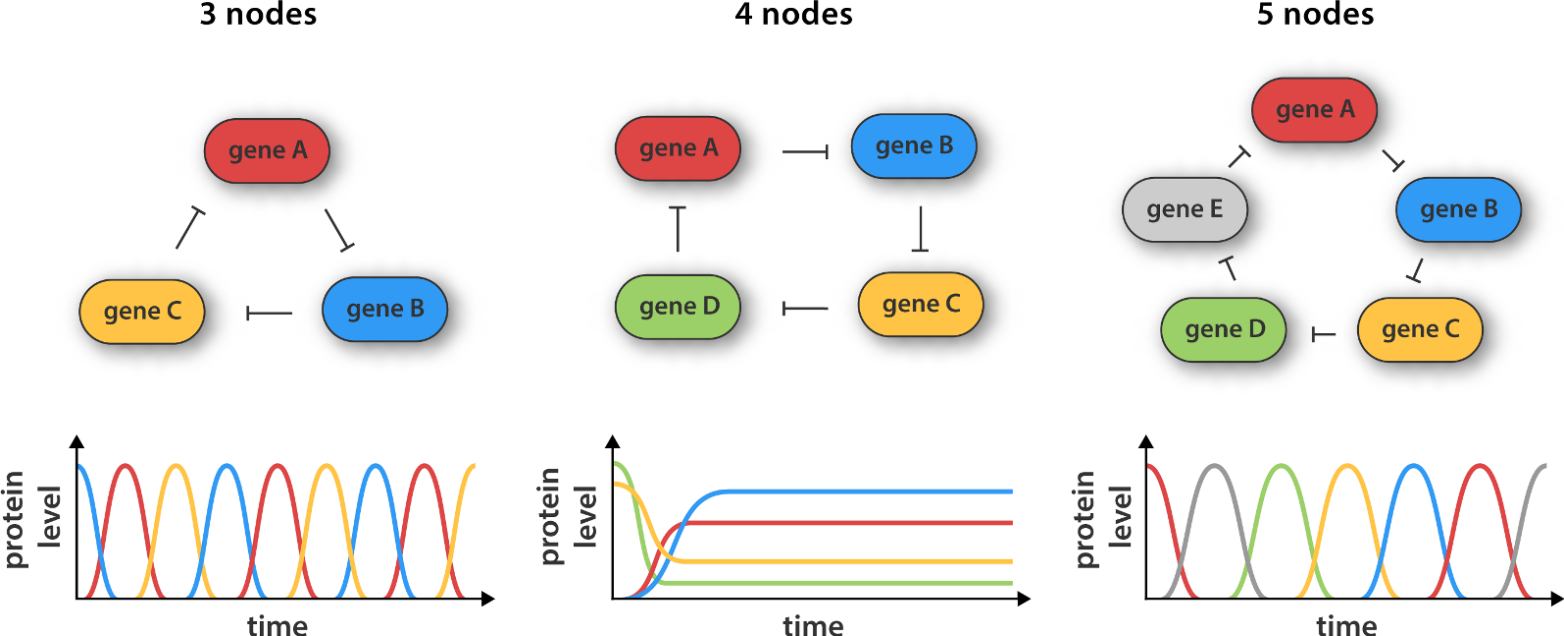
Synthetic gene networks containing three, four and five genes. The genes in each circuit (top) are translated into protein products, with each protein product repressing the activity of another gene in the network (as indicated by the arrows). Theory predicts that cyclic networks of genes display oscillatory behavior when the number of nodes in the network is odd. Niederholtmeyer et al. found that a circuit consisting of three genes gave rise to well-defined oscillations with a period of up to 8 hr, and that a circuit containing five genes oscillated with a period of 19 hours. In contrast, and in line with theoretical predictions, a network consisting of four nodes did not oscillate: instead it reached a steady state where the activity of all the genes was constant over time.

Now Maerkl and co-workers – who are based at the École Polytechnique Fédérale de Lausanne and the California Institute of Technology – have made ring architectures containing three, four and five genes (Figure 1). They built their prototype genetic circuits in a cell-free system by combining microfluidic flow reactors with extracts from *E. coli bacteria* (Niederholtmeyer et al, 2013; Noireaux et al., 2003). The major advantage of this approach is that it significantly decreases the time taken for each design-build-test cycle because it removes the need for various laborious tasks, such as molecular cloning and collecting measurements from individual cells.

Using this strategy Niederholtmeyer, Sun et al. were able to confirm the prediction that oscillators with three or five nodes are able to generate oscillations, whereas oscillators with four nodes are not. The period of the five-node oscillator is about twice as long as the three-node oscillator, indicating that cells can tune the periodicity of signals by increasing the complexity of their genetic circuits. Next, the researchers transferred their prototyped designs to living *E. coli* cells and showed that the oscillation period in cells matched the oscillation period in the cell-free systems. This is an important result as it shows that cell-free systems can be used to accurately capture the behavior of cells, which paves the way for researchers to use synthetic biology approaches in cell-free systems to explore the complex regulatory mechanisms that operate inside cells (van Roekel et al., 2015). The latest work should also greatly speed up the construction of complex new gene networks in bacteria, which could have applications in biofuel production, medical diagnosis and experiments to explore the ways that cells process information.
